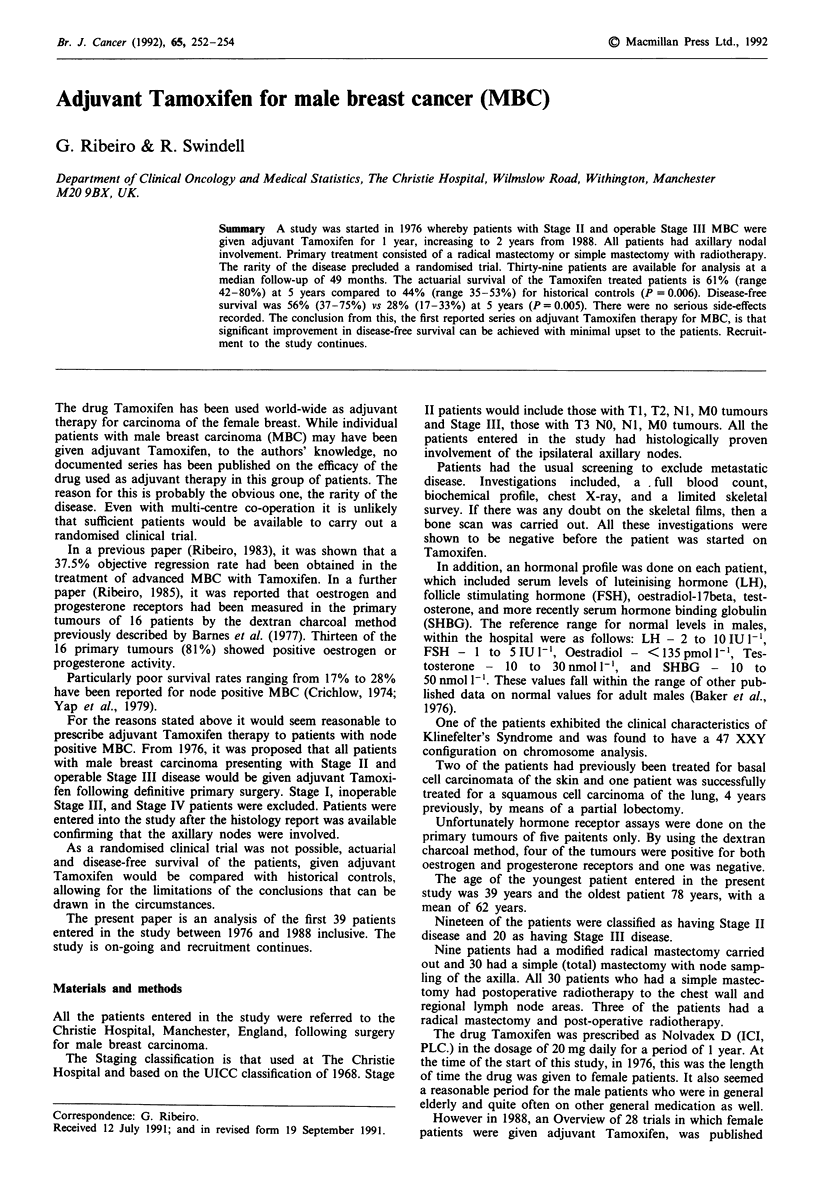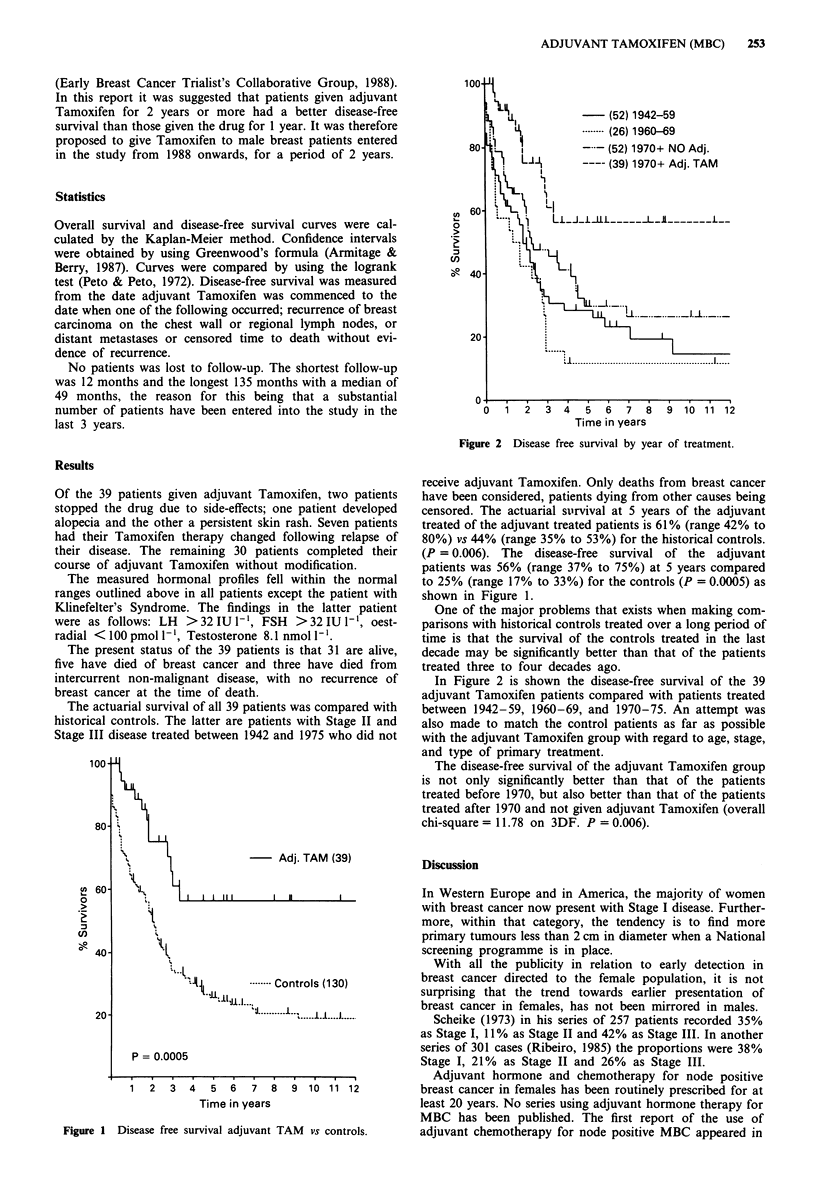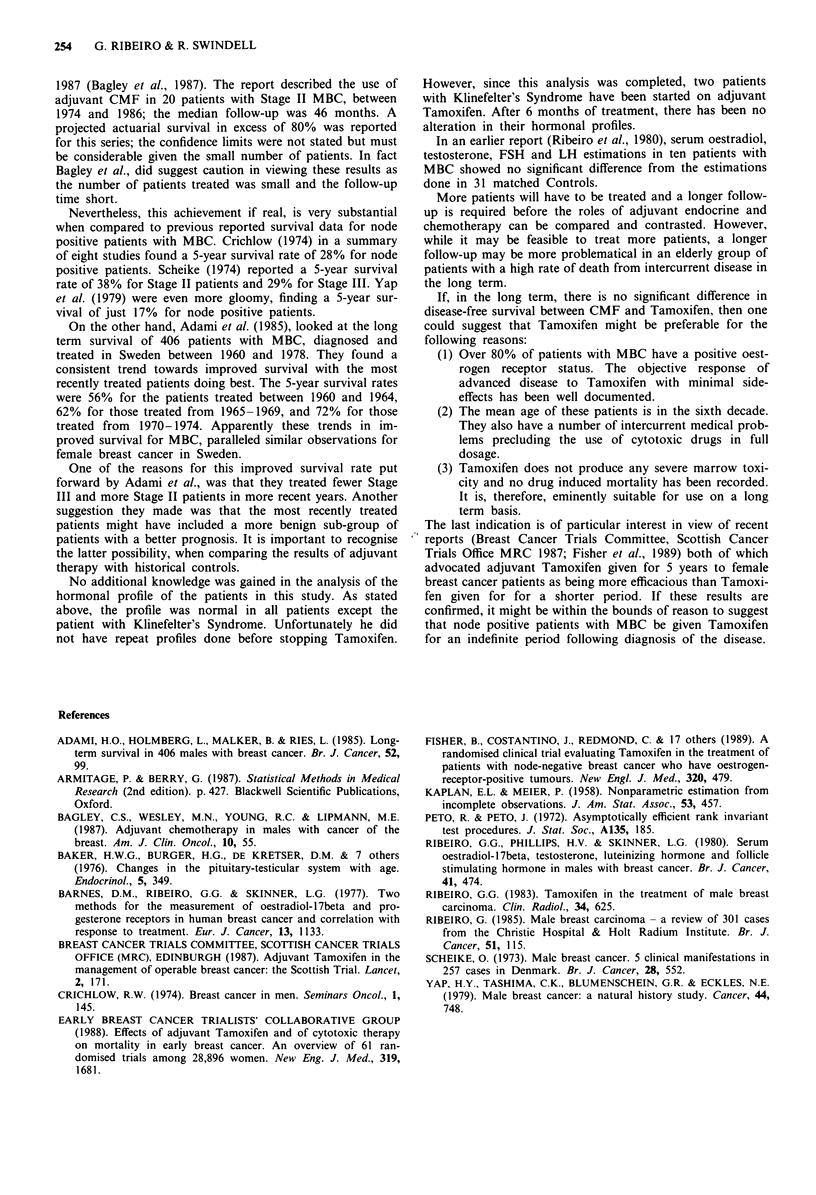# Adjuvant tamoxifen for male breast cancer (MBC).

**DOI:** 10.1038/bjc.1992.50

**Published:** 1992-02

**Authors:** G. Ribeiro, R. Swindell

**Affiliations:** Department of Clinical Oncology and Medical Statistics, Christie Hospital, Withington, Manchester, UK.

## Abstract

A study was started in 1976 whereby patients with Stage II and operable Stage III MBC were given adjuvant Tamoxifen for 1 year, increasing to 2 years from 1988. All patients had axillary nodal involvement. Primary treatment consisted of a radical mastectomy or simple mastectomy with radiotherapy. The rarity of the disease precluded a randomised trial. Thirty-nine patients are available for analysis at a median follow-up of 49 months. The actuarial survival of the Tamoxifen treated patients is 61% (range 42-80%) at 5 years compared to 44% (range 35-53%) for historical controls (P = 0.006). Disease-free survival was 56% (37-75%) vs 28% (17-33%) at 5 years (P = 0.005). There were no serious side-effects recorded. The conclusion from this, the first reported series on adjuvant Tamoxifen therapy for MBC, is that significant improvement in disease-free survival can be achieved with minimal upset to the patients. Recruitment to the study continues.


Br. J. Cancer (1992), 65, 252-254 © Macmillan Press Ltd., 1992

### Adjuvant Tamoxifen for male breast cancer (MBC)

G. Ribeiro & R. Swindell

*Department of Clinical Oncology and Medical Statistics, The Christie Hospital, Wilmslow Road, Withington, Manchester M20 9BX, UK.*

**Summary** A study was started in 1976 whereby patients with Stage II and operable Stage III MBC were given adjuvant Tamoxifen for 1 year, increasing to 2 years from 1988. All patients had axillary nodal involvement. Primary treatment consisted of a radical mastectomy or simple mastectomy with radiotherapy. The rarity of the disease precluded a randomised trial. Thirty-nine patients are available for analysis at a median follow-up of 49 months. The actuarial survival of the Tamoxifen treated patients is 61% (range 42–80%) at 5 years compared to 44% (range 35–53%) for historical controls ($P = 0.006$). Disease-free survival was 56% (37–75%) *vs* 28% (17–33%) at 5 years ($P = 0.005$). There were no serious side-effects recorded. The conclusion from this, the first reported series on adjuvant Tamoxifen therapy for MBC, is that significant improvement in disease-free survival can be achieved with minimal upset to the patients. Recruitment to the study continues.

The drug Tamoxifen has been used world-wide as adjuvant therapy for carcinoma of the female breast. While individual patients with male breast carcinoma (MBC) may have been given adjuvant Tamoxifen, to the authors' knowledge, no documented series has been published on the efficacy of the drug used as adjuvant therapy in this group of patients. The reason for this is probably the obvious one, the rarity of the disease. Even with multi-centre co-operation it is unlikely that sufficient patients would be available to carry out a randomised clinical trial.

In a previous paper (Ribeiro, 1983), it was shown that a 37.5% objective regression rate had been obtained in the treatment of advanced MBC with Tamoxifen. In a further paper (Ribeiro, 1985), it was reported that oestrogen and progesterone receptors had been measured in the primary tumours of 16 patients by the dextran charcoal method previously described by Barnes *et al.* (1977). Thirteen of the 16 primary tumours (81%) showed positive oestrogen or progesterone activity.

Particularly poor survival rates ranging from 17% to 28% have been reported for node positive MBC (Crichlow, 1974; Yap *et al.*, 1979).

For the reasons stated above it would seem reasonable to prescribe adjuvant Tamoxifen therapy to patients with node positive MBC. From 1976, it was proposed that all patients with male breast carcinoma presenting with Stage II and operable Stage III disease would be given adjuvant Tamoxifen following definitive primary surgery. Stage I, inoperable Stage III, and Stage IV patients were excluded. Patients were entered into the study after the histology report was available confirming that the axillary nodes were involved.

As a randomised clinical trial was not possible, actuarial and disease-free survival of the patients, given adjuvant Tamoxifen would be compared with historical controls, allowing for the limitations of the conclusions that can be drawn in the circumstances.

The present paper is an analysis of the first 39 patients entered in the study between 1976 and 1988 inclusive. The study is on-going and recruitment continues.

### Materials and methods

All the patients entered in the study were referred to the Christie Hospital, Manchester, England, following surgery for male breast carcinoma.

The Staging classification is that used at The Christie Hospital and based on the UICC classification of 1968. Stage II patients would include those with T1, T2, N1, M0 tumours and Stage III, those with T3 N0, N1, M0 tumours. All the patients entered in the study had histologically proven involvement of the ipsilateral axillary nodes.

Patients had the usual screening to exclude metastatic disease. Investigations included, a full blood count, biochemical profile, chest X-ray, and a limited skeletal survey. If there was any doubt on the skeletal films, then a bone scan was carried out. All these investigations were shown to be negative before the patient was started on Tamoxifen.

In addition, an hormonal profile was done on each patient, which included serum levels of luteinising hormone (LH), follicle stimulating hormone (FSH), oestradiol-17beta, testosterone, and more recently serum hormone binding globulin (SHBG). The reference range for normal levels in males, within the hospital were as follows: LH – 2 to 10 IU $l^{-1}$, FSH – 1 to 5 IU $l^{-1}$, Oestradiol – $<$135 pmol $l^{-1}$, Testosterone – 10 to 30 nmol $l^{-1}$, and SHBG – 10 to 50 nmol $l^{-1}$. These values fall within the range of other published data on normal values for adult males (Baker *et al.*, 1976).

One of the patients exhibited the clinical characteristics of Klinefelter's Syndrome and was found to have a 47 XXY configuration on chromosome analysis.

Two of the patients had previously been treated for basal cell carcinomata of the skin and one patient was successfully treated for a squamous cell carcinoma of the lung, 4 years previously, by means of a partial lobectomy.

Unfortunately hormone receptor assays were done on the primary tumours of five paitents only. By using the dextran charcoal method, four of the tumours were positive for both oestrogen and progesterone receptors and one was negative.

The age of the youngest patient entered in the present study was 39 years and the oldest patient 78 years, with a mean of 62 years.

Nineteen of the patients were classified as having Stage II disease and 20 as having Stage III disease.

Nine patients had a modified radical mastectomy carried out and 30 had a simple (total) mastectomy with node sampling of the axilla. All 30 patients who had a simple mastectomy had postoperative radiotherapy to the chest wall and regional lymph node areas. Three of the patients had a radical mastectomy and post-operative radiotherapy.

The drug Tamoxifen was prescribed as Nolvadex D (ICI, PLC.) in the dosage of 20 mg daily for a period of 1 year. At the time of the start of this study, in 1976, this was the length of time the drug was given to female patients. It also seemed a reasonable period for the male patients who were in general elderly and quite often on other general medication as well.

However in 1988, an Overview of 28 trials in which female patients were given adjuvant Tamoxifen, was published

Correspondence: G. Ribeiro.
Received 12 July 1991; and in revised form 19 September 1991.

(Early Breast Cancer Trialist's Collaborative Group, 1988). In this report it was suggested that patients given adjuvant Tamoxifen for 2 years or more had a better disease-free survival than those given the drug for 1 year. It was therefore proposed to give Tamoxifen to male breast patients entered in the study from 1988 onwards, for a period of 2 years.

### Statistics

Overall survival and disease-free survival curves were calculated by the Kaplan-Meier method. Confidence intervals were obtained by using Greenwood's formula (Armitage & Berry, 1987). Curves were compared by using the logrank test (Peto & Peto, 1972). Disease-free survival was measured from the date adjuvant Tamoxifen was commenced to the date when one of the following occurred; recurrence of breast carcinoma on the chest wall or regional lymph nodes, or distant metastases or censored time to death without evidence of recurrence.

No patients was lost to follow-up. The shortest follow-up was 12 months and the longest 135 months with a median of 49 months, the reason for this being that a substantial number of patients have been entered into the study in the last 3 years.

### Results

Of the 39 patients given adjuvant Tamoxifen, two patients stopped the drug due to side-effects; one patient developed alopecia and the other a persistent skin rash. Seven patients had their Tamoxifen therapy changed following relapse of their disease. The remaining 30 patients completed their course of adjuvant Tamoxifen without modification.

The measured hormonal profiles fell within the normal ranges outlined above in all patients except the patient with Klinefelter's Syndrome. The findings in the latter patient were as follows: LH $>32\,IU\,l^{-1}$, FSH $>32\,IU\,l^{-1}$, oestradial $<100\,pmol\,l^{-1}$, Testosterone $8.1\,nmol\,l^{-1}$.

The present status of the 39 patients is that 31 are alive, five have died of breast cancer and three have died from intercurrent non-malignant disease, with no recurrence of breast cancer at the time of death.

The actuarial survival of all 39 patients was compared with historical controls. The latter are patients with Stage II and Stage III disease treated between 1942 and 1975 who did not

**Figure 1** Disease free survival adjuvant TAM *vs* controls.

**Figure 2** Disease free survival by year of treatment.

receive adjuvant Tamoxifen. Only deaths from breast cancer have been considered, patients dying from other causes being censored. The actuarial survival at 5 years of the adjuvant treated of the adjuvant treated patients is 61% (range 42% to 80%) *vs* 44% (range 35% to 53%) for the historical controls. ($P = 0.006$). The disease-free survival of the adjuvant patients was 56% (range 37% to 75%) at 5 years compared to 25% (range 17% to 33%) for the controls ($P = 0.0005$) as shown in Figure 1.

One of the major problems that exists when making comparisons with historical controls treated over a long period of time is that the survival of the controls treated in the last decade may be significantly better than that of the patients treated three to four decades ago.

In Figure 2 is shown the disease-free survival of the 39 adjuvant Tamoxifen patients compared with patients treated between 1942–59, 1960–69, and 1970–75. An attempt was also made to match the control patients as far as possible with the adjuvant Tamoxifen group with regard to age, stage, and type of primary treatment.

The disease-free survival of the adjuvant Tamoxifen group is not only significantly better than that of the patients treated before 1970, but also better than that of the patients treated after 1970 and not given adjuvant Tamoxifen (overall chi-square = 11.78 on 3DF. $P = 0.006$).

### Discussion

In Western Europe and in America, the majority of women with breast cancer now present with Stage I disease. Furthermore, within that category, the tendency is to find more primary tumours less than 2 cm in diameter when a National screening programme is in place.

With all the publicity in relation to early detection in breast cancer directed to the female population, it is not surprising that the trend towards earlier presentation of breast cancer in females, has not been mirrored in males.

Scheike (1973) in his series of 257 patients recorded 35% as Stage I, 11% as Stage II and 42% as Stage III. In another series of 301 cases (Ribeiro, 1985) the proportions were 38% Stage I, 21% as Stage II and 26% as Stage III.

Adjuvant hormone and chemotherapy for node positive breast cancer in females has been routinely prescribed for at least 20 years. No series using adjuvant hormone therapy for MBC has been published. The first report of the use of adjuvant chemotherapy for node positive MBC appeared in

1987 (Bagley *et al.*, 1987). The report described the use of adjuvant CMF in 20 patients with Stage II MBC, between 1974 and 1986; the median follow-up was 46 months. A projected actuarial survival in excess of 80% was reported for this series; the confidence limits were not stated but must be considerable given the small number of patients. In fact Bagley *et al.*, did suggest caution in viewing these results as the number of patients treated was small and the follow-up time short.

Nevertheless, this achievement if real, is very substantial when compared to previous reported survival data for node positive patients with MBC. Crichlow (1974) in a summary of eight studies found a 5-year survival rate of 28% for node positive patients. Scheike (1974) reported a 5-year survival rate of 38% for Stage II patients and 29% for Stage III. Yap *et al.* (1979) were even more gloomy, finding a 5-year survival of just 17% for node positive patients.

On the other hand, Adami *et al.* (1985), looked at the long term survival of 406 patients with MBC, diagnosed and treated in Sweden between 1960 and 1978. They found a consistent trend towards improved survival with the most recently treated patients doing best. The 5-year survival rates were 56% for the patients treated between 1960 and 1964, 62% for those treated from 1965–1969, and 72% for those treated from 1970–1974. Apparently these trends in improved survival for MBC, paralleled similar observations for female breast cancer in Sweden.

One of the reasons for this improved survival rate put forward by Adami *et al.*, was that they treated fewer Stage III and more Stage II patients in more recent years. Another suggestion they made was that the most recently treated patients might have included a more benign sub-group of patients with a better prognosis. It is important to recognise the latter possibility, when comparing the results of adjuvant therapy with historical controls.

No additional knowledge was gained in the analysis of the hormonal profile of the patients in this study. As stated above, the profile was normal in all patients except the patient with Klinefelter's Syndrome. Unfortunately he did not have repeat profiles done before stopping Tamoxifen. However, since this analysis was completed, two patients with Klinefelter's Syndrome have been started on adjuvant Tamoxifen. After 6 months of treatment, there has been no alteration in their hormonal profiles.

In an earlier report (Ribeiro *et al.*, 1980), serum oestradiol, testosterone, FSH and LH estimations in ten patients with MBC showed no significant difference from the estimations done in 31 matched Controls.

More patients will have to be treated and a longer follow-up is required before the roles of adjuvant endocrine and chemotherapy can be compared and contrasted. However, while it may be feasible to treat more patients, a longer follow-up may be more problematical in an elderly group of patients with a high rate of death from intercurrent disease in the long term.

If, in the long term, there is no significant difference in disease-free survival between CMF and Tamoxifen, then one could suggest that Tamoxifen might be preferable for the following reasons:

(1) Over 80% of patients with MBC have a positive oestrogen receptor status. The objective response of advanced disease to Tamoxifen with minimal side-effects has been well documented.
(2) The mean age of these patients is in the sixth decade. They also have a number of intercurrent medical problems precluding the use of cytotoxic drugs in full dosage.
(3) Tamoxifen does not produce any severe marrow toxicity and no drug induced mortality has been recorded. It is, therefore, eminently suitable for use on a long term basis.

The last indication is of particular interest in view of recent reports (Breast Cancer Trials Committee, Scottish Cancer Trials Office MRC 1987; Fisher *et al.*, 1989) both of which advocated adjuvant Tamoxifen given for 5 years to female breast cancer patients as being more efficacious than Tamoxifen given for for a shorter period. If these results are confirmed, it might be within the bounds of reason to suggest that node positive patients with MBC be given Tamoxifen for an indefinite period following diagnosis of the disease.

### References

ADAMI, H.O., HOLMBERG, L., MALKER, B. & RIES, L. (1985). Long-term survival in 406 males with breast cancer. *Br. J. Cancer*, **52**, 99.

ARMITAGE, P. & BERRY, G. (1987). *Statistical Methods in Medical Research* (2nd edition). p. 427. Blackwell Scientific Publications, Oxford.

BAGLEY, C.S., WESLEY, M.N., YOUNG, R.C. & LIPMANN, M.E. (1987). Adjuvant chemotherapy in males with cancer of the breast. *Am. J. Clin. Oncol.*, **10**, 55.

BAKER, H.W.G., BURGER, H.G., DE KRETSER, D.M. & 7 others (1976). Changes in the pituitary-testicular system with age. *Endocrinol.*, **5**, 349.

BARNES, D.M., RIBEIRO, G.G. & SKINNER, L.G. (1977). Two methods for the measurement of oestradiol-17beta and progesterone receptors in human breast cancer and correlation with response to treatment. *Eur. J. Cancer*, **13**, 1133.

BREAST CANCER TRIALS COMMITTEE, SCOTTISH CANCER TRIALS OFFICE (MRC), EDINBURGH (1987). Adjuvant Tamoxifen in the management of operable breast cancer: the Scottish Trial. *Lancet*, **2**, 171.

CRICHLOW, R.W. (1974). Breast cancer in men. *Seminars Oncol.*, **1**, 145.

EARLY BREAST CANCER TRIALISTS' COLLABORATIVE GROUP (1988). Effects of adjuvant Tamoxifen and of cytotoxic therapy on mortality in early breast cancer. An overview of 61 randomised trials among 28,896 women. *New Eng. J. Med.*, **319**, 1681.

FISHER, B., COSTANTINO, J., REDMOND, C. & 17 others (1989). A randomised clinical trial evaluating Tamoxifen in the treatment of patients with node-negative breast cancer who have oestrogen-receptor-positive tumours. *New Engl. J. Med.*, **320**, 479.

KAPLAN, E.L. & MEIER, P. (1958). Nonparametric estimation from incomplete observations. *J. Am. Stat. Assoc.*, **53**, 457.

PETO, R. & PETO, J. (1972). Asymptotically efficient rank invariant test procedures. *J. Stat. Soc.*, **A135**, 185.

RIBEIRO, G.G., PHILLIPS, H.V. & SKINNER, L.G. (1980). Serum oestradiol-17beta, testosterone, luteinizing hormone and follicle stimulating hormone in males with breast cancer. *Br. J. Cancer*, **41**, 474.

RIBEIRO, G.G. (1983). Tamoxifen in the treatment of male breast carcinoma. *Clin. Radiol.*, **34**, 625.

RIBEIRO, G. (1985). Male breast carcinoma – a review of 301 cases from the Christie Hospital & Holt Radium Institute. *Br. J. Cancer*, **51**, 115.

SCHEIKE, O. (1973). Malc breast cancer. 5 clinical manifestations in 257 cases in Denmark. *Br. J. Cancer*, **28**, 552.

YAP, H.Y., TASHIMA, C.K., BLUMENSCHEIN, G.R. & ECKLES, N.E. (1979). Male breast cancer: a natural history study. *Cancer*, **44**, 748.